# Quantitation of Gene Expression in Formaldehyde-Fixed and Fluorescence-Activated Sorted Cells

**DOI:** 10.1371/journal.pone.0073849

**Published:** 2013-09-02

**Authors:** Julia N. Russell, Janice E. Clements, Lucio Gama

**Affiliations:** Department of Molecular and Comparative Pathobiology, Johns Hopkins University School of Medicine, Baltimore, Maryland, United States of America; George Mason University, United States of America

## Abstract

Fluorescence-activated cell sorting (FACS) is a sensitive and valuable technique to characterize cellular subpopulations and great advances have been made using this approach. Cells are often fixed with formaldehyde prior to the sorting process to preserve cell morphology and maintain the expression of surface molecules, as well as to ensure safety in the sorting of infected cells. It is widely recognized that formaldehyde fixation alters RNA and DNA structure and integrity, thus analyzing gene expression in these cells has been difficult. We therefore examined the effects of formaldehyde fixation on the stability and quantitation of nucleic acids in cell lines, primary leukocytes and also cells isolated from SIV-infected pigtailed macaques. We developed a method to extract RNA from fixed cells that yielded the same amount of RNA as our common method of RNA isolation from fresh cells. Quantitation of RNA by RT-qPCR in fixed cells was not always comparable with that in unfixed cells. In comparison, when RNA was measured by the probe-based NanoString system, there was no significant difference in RNA quantitation. In addition, we demonstrated that quantitation of proviral DNA in fixed cells by qPCR is comparable to that in unfixed cells when normalized by a single-copy cellular gene. These results provide a systematic procedure to quantitate gene expression in cells that have been fixed with formaldehyde and sorted by FACS.

## Introduction

Phenotypic and functional characterization of cellular subpopulations has been substantially advanced since the advent of fluorescence-activated cell sorting (FACS). Characterization of diverse cell populations by FACS can be followed by molecular analysis of homogeneously sorted subsets, which represents an important step in elucidating their role in homeostasis as well as disease pathogenesis.

Molecular analysis of live, sorted cells, however, can be hindered by a variety of factors. Specifically, certain primary cell types, such as blood monocytes, undergo changes once isolated from whole blood and rapidly lose viability [1], [2]. The staining and sorting process often takes several hours, and cell morphology and integrity can be compromised unless the cells are fixed. In addition, samples acquired from individuals infected with highly infectious pathogens, such as HIV or HCV, are often restricted to facilities with biosafety containment, and usually require fixation if they are transported, processed or analyzed outside of an appropriate biosafety laboratory. Therefore, cell fixation would eliminate potential barriers to studying cell subpopulations by FACS. Here we examined whether quantitation of nucleic acids in fixed cells correlated with quantitation in unfixed cells.

Although numerous fixatives exist, fixation with a weak formaldehyde solution is routinely performed on cells prepared for FACS analysis [3]–[5]. Formaldehyde fixation cross-links nucleic acids to proteins and causes chemical modifications of RNA, DNA and protein, which can compromise nucleic acid integrity, limiting the efficiency of isolation, detection, and accurate quantitation. Previous studies have demonstrated the analysis and quantitation of gene expression by RT-PCR from formalin-fixed, paraffin embedded tissue (FFPE) [6]–[10]. It also has been shown that quantitation of RNA expression is similar to that before fixation under certain conditions [11]. A number of factors affect RNA detection by RT-PCR in fixed tissue, including amplicon size and the time between fixation and nucleic acid isolation [11]–[14]. A recent study by Reis et al. [15] showed that the NanoString nCounter**®** gene expression system, a probe-based technology, is more accurate than RT-qPCR in quantitating gene expression in FFPE tissues. To our knowledge, there have been no reports on comparing RNA isolation and quantitation from fresh and fixed cells prepared for FACS analysis.

In this study, we developed a method for RNA isolation from formaldehyde-fixed cells and compared RNA quantitation in fresh and fixed cells using RT-qPCR and NanoString. Additionally, we sorted CD3^+^ and CD14^+^ cells from fresh and fixed human peripheral blood mononuclear cells (PBMCs) to assess the factors of time and sorting on transcriptome analysis. Finally, to address the effects of formaldehyde fixation on DNA, we compared the number of proviral DNA copies by qPCR from fresh and fixed PBMCs isolated from pigtailed macaques infected with simian immunodeficiency virus (SIV). We demonstrate that RNA isolation from fixed cells is comparable to unfixed in yield of RNA, and that quantitation of mRNA transcripts by NanoString, but not by RT-qPCR, is equally efficient in fixed and unfixed cells. Fixation of certain primary cells preserves their phenotype and may provide a better approach to their characterization. Further, SIV DNA levels in fixed and unfixed cells were comparable when normalized to a cellular gene. Thus, we demonstrate that analyses of both RNA and DNA in fixed cells provide results comparable to that of unfixed cells.

## Methods

### Ethics Statement and Animal Work

Animal studies were approved by the Johns Hopkins University Institutional Animal Care and Use Committee and conducted in accordance with the Weatherall Report, the Guide for the Care and Use of Laboratory Animals, and the USDA Animal Welfare Act. Three juvenile pigtailed macaques (*Macaca nemestrina*) were intravenously inoculated with SIV/DeltaB670 and SIV/17E-Fr as previously described [16]. They were treated once daily with oral 25 mg/kg fisetin, a neuroprotective compound, starting on day 12 post-inoculation, which we did not predict would interfere with our results. Animals were monitored twice daily by veterinarians or trained technicians throughout the study for clinical signs of disease, such as weight loss, intractable diarrhea and opportunistic infection, in order that early endpoint could be performed if necessary. Macaques were housed in Johns Hopkins University facilities that are fully accredited by the Association for the Assessment and Accreditation of Laboratory Animal Care, International (AAALAC), and fed a balanced commercial macaque chow (Purina Mills, Gray Summit, MO, USA). All animals received environmental enrichment including manipulanda and novel foodstuffs throughout the study, and were housed in groups prior to infection and in cages providing 6 square feet of space and visual and auditory contact of conspecifics following infection. Individual housing was performed following infection to ensure each animal received the appropriate drug dosage and prevent horizontal transmission of infection after the initial inoculation date. We scheduled necropsies at pre-specified timepoints during terminal infection (between days 83–85 post-inoculation) because these timepoints occur prior to the onset of clinical signs in our highly consistent SIV/pigtailed macaque model [17]. For necropsy, animals were euthanized using an overdose of sodium pentobarbital while under ketamine sedation (10 mg/kg intramuscular injection) prior to perfusion with PBS to remove blood from tissues as described by Zink et al. [16].

### PBMC Isolation

PBMCs were obtained from whole blood harvested from three juvenile pigtailed macaques (*Macaca nemestrina*) at necropsy and PBMCs were isolated using a Percoll (GE Healthcare, Pittsburgh, PA, USA) gradient according to the manufacturer’s protocol.

Human PBMCs were obtained from anonymous, healthy donors of the New York Blood Center. Cells were isolated using a Ficoll-Paque (GE Healthcare) gradient according to the manufacturer’s protocol.

### Cell Culture

K562 and CEMx174 cell lines were obtained from ATCC (Manassas, VA, USA). Cells were cultured in RPMI with 10% FBS, 2 mM L-glutamine, 1 mM HEPES and 2 mg/ml gentamicin.

### Fixation

All cell lines and primary cells were fixed in 20 volumes of BD FACS Lysing Solution (BD Bioscience, San Jose, CA, USA), which contains <1.5% formaldehyde at 1X concentration, for 10 minutes.

### FACS Sorting

Human PBMCs were stained with anti-CD3 (PE; clone SP34; BD Bioscience) and anti-CD14 (FITC; clone M5E2; BD Bioscience) for 20 minutes at room temperature. Half of the cells were kept on ice and the other half was fixed as described above. Both fixed and unfixed cells were sorted according to their surface marker on a BDFACSAria II cytometer (BD Bioscience). In addition, cells from the same PBMC sample were fixed, incubated at 4°C for 24 hours and then sorted for CD3^+^ and CD14^+^ expression.

### Total RNA Isolation

Total RNA was isolated from fresh cells using the RNeasy Plus Mini kit (Qiagen, Valencia, CA, USA) following the manufacturer’s protocol. Between column washes, an on-column DNase digestion was performed using the RNase-free DNase kit (Qiagen), with the addition of four units of TURBO DNase (Life Technologies, Carlsbad, CA, USA) to the enzyme mix.

Total RNA was isolated from fixed cells using reagents from the RNeasy FFPE kit (Qiagen) and the RNeasy Plus Mini Kit (Qiagen), following a modified version of the manufacturers’ protocols. Cells were initially resuspended in 240 µl of Buffer PKD. Following the addition of 10 µl of proteinase K, samples were incubated at 56°C for 15 minutes, then at 80°C for 15 minutes. Five hundred microliters of Buffer RBC was added and then samples were passed through the gDNA Eliminator column. After the addition of 1200 µl of 100% ethanol to the flow-through, samples were passed through the RNeasy MiniElute spin column. Samples were washed with Buffers RW1 and RPE and eluted with RNase-free water following the manufacturer’s protocol. An on-column DNase digestion was performed as described above for RNA isolation from fresh cells. RNA isolated from sorted cells was used as samples for the probe-based NanoString system and therefore did not undergo DNase treatment.

Assessment of RNA quality was performed using the Agilent 2100 Bioanalyzer (Agilent Technologies, Santa Clara, CA, USA) by the Johns Hopkins Deep Sequencing & Microarray Core at the Johns Hopkins University School of Medicine, Baltimore, MD, USA.

### RNA Quantification by RT-qPCR

Superscript II enzyme (Life Technologies) was used to synthesize cDNA from 1 µg (cell lines) or 100 ng (PBMCs) of total RNA. PCR reactions were performed using QuantiTect Multiplex PCR Mix (Qiagen) and primers and probes specific to genes evaluated (Table S2). Reactions were run in duplicate wells together with no-reverse transcriptase controls using the following protocol: step 1: 95° for 10 min, step 2: 95° for 15 seconds, 55° for 15 seconds, 60° for 30 seconds, with step 2 repeated 45 times.

### NanoString nCounter® Gene Expression System

The nCounter**®** Human Reference GX kit (NanoString Technologies, Seattle, WA, USA) was used to measure the expression of 18 human reference genes in our RNA samples (Table S3). Following hybridization, transcripts were quantitated using the nCounter**®** Digital Analyzer [18]. Data were normalized for assay efficiency by multiplying each count by a positive normalization factor obtained for each sample. Samples were run by the Johns Hopkins Deep Sequencing & Microarray Core. To remove background, the highest negative control value for each sample was subtracted from each count value of that sample. Following background subtraction, any negative count values were considered as 0. Counts were normalized to total RNA loaded. Raw data can be found at the Gene Expression Omnibus database under the name of the corresponding author (http://www.ncbi.nlm.nih.gov/geo/).

### Total DNA Isolation

Total DNA was isolated from macaque PBMCs using the DNeasy Blood & Tissue Kit (Qiagen) following the manufacturer’s protocol.

Total DNA was isolated from fixed cells using the QIAamp DNA FFPE Tissue Kit (Qiagen). Cells were resuspended in 180 µl of Buffer ATL and 20 µl of proteinase K was added. Cells were incubated at 56°C for 1 hour, then at 90°C for 1 hour. Following the addition of 200 µl of Buffer AL and 200 µl of 100% ethanol, samples were passed through the QIAamp MiniElute column, washed and eluted following the manufacturer’s instructions.

### Viral and Cellular DNA Quantification

SIV *gag* and IFNβ DNA were measured from 200 ng of total DNA by qPCR using QuantiTect Multiplex PCR Mix (Qiagen) and gene specific primers and probes (Table S2). Reactions were run in triplicate wells using the following protocol: step 1: 95° for 10 min, step 2: 95° for 15 seconds, 55° for 15 seconds, 60° for 30 seconds, with step 2 repeated 45 times. Copy numbers were obtained by extrapolation from standard curves [19].

### Statistical Analysis

Differences in RNA quantitation between unfixed and fixed samples by qPCR were analyzed by a one-sample, two-tailed t test with theoretical mean set to 0. Correlations between transcript counts for unfixed and fixed samples obtained using NanoString were determined by a Pearson correlation analysis. Differences in DNA quantitation between unfixed and fixed macaque PBMCs were analyzed by Wilcoxon matched-pairs rank test. RNA and DNA yields were compared between unfixed and fixed samples by a two-tailed, paired t test. Statistics were performed using Prism software (GraphPad Software, La Jolla, CA, USA).

## Results

### RNA Quantitation using RT-qPCR

To determine whether there was variability in the quantitation of RNA between fixed and unfixed cells, we isolated total RNA from two cell lines and primary cells, and measured four genes by RT-qPCR. In all experiments, half of the cells were fixed using the BD FACS Lysing Solution, which at 1X concentration is equivalent to a <1.5% formaldehyde solution. We then performed two different protocols for RNA isolation of unfixed and fixed cells (see Methods section). Quantitation of RNA yield by absorbance analysis demonstrated no difference in yield between protocols when isolating RNA from cells lines K562 and CEMx174, and human PBMCs (Figure S1).

**Figure 1:**
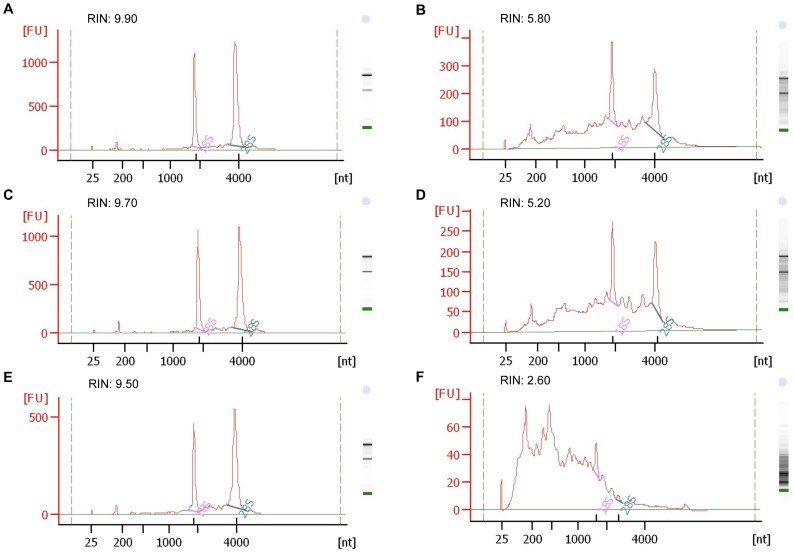
Analysis of RNA integrity in unfixed and fixed cells. RNA integrity analysis of RNA isolated from unfixed and fixed samples was performed using Agilent 2100 Bioanalyzer. Representative electropherograms for unfixed (A, C, E) and fixed (B, D, F) samples of each cell type, K562 (A, B), CEMx174 (C, D), human PBMCs (E, F), are shown. The RNA Integrity Number (RIN) is shown for each sample.

A RNA integrity number (RIN) was then determined for each sample using an established algorithm following sample assessment in an automated electrophoresis system (Agilent 2100 Bioanalyzer) [20]. We observed significant RNA degradation in fixed samples of all cell types as seen in representative electropherograms and corresponding RIN values (Figure 1). The mean RIN for all unfixed samples was 9.8 compared to a mean RIN of 5.8 for fixed samples. The data also showed that RNA degradation was more significant in human PBMCs than in cell lines (Figures 1B, D and F), suggesting that RNA in primary cells may be more sensitive to the fixation process.

**Figure 2:**
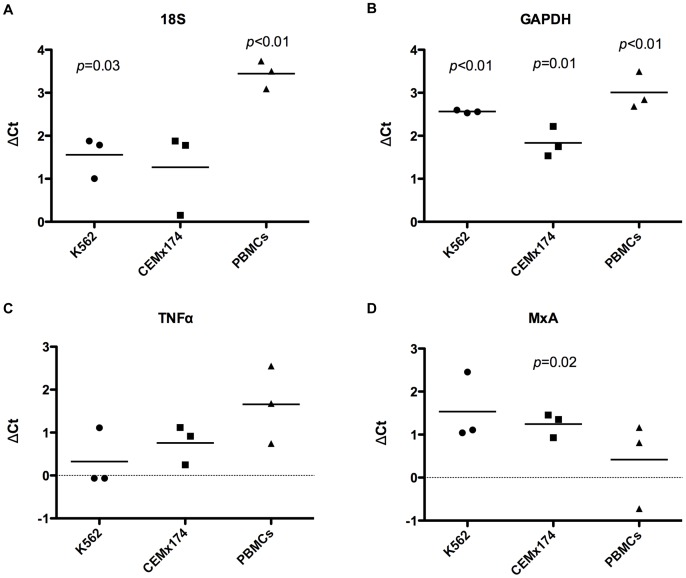
Comparison of mRNA quantitation by RT-qPCR between unfixed and fixed cells. 18S (A), GAPDH (B), TNFα (C) and MxA (D) mRNA quantitation by RT-qPCR were compared between unfixed and fixed samples of two cell lines, K562 and CEMx174, and human PBMCs. Individual points represent the difference of Ct values between unfixed and fixed samples for each gene obtained from one experiment (ΔCt = Fixed Ct – Unfixed Ct). Three biological replicates are shown for each sample. *p* values were calculated by a one-sample, two-tailed t test comparing the average ΔCt to a theoretical mean set to zero. Statistically significant *p* values are shown.

To evaluate whether fixation of cells altered the quantitation of RNA, we performed RT-qPCR for 18S, GAPDH, TNFα and MxA in K562 and CEMx174 cell lines and PBMCs. In K562 cells and PBMCs, the levels of 18S and GAPDH were significantly higher in RNA isolated from fresh cells (Figures 2A and 2B). In contrast, quantitation of TNFα and MxA was not significantly different between unfixed and fixed samples for most cell types (Figures 2C and 2D).

We compared the amplicon size used in the quantitation of the 18S and GAPDH RNAs (>100 bp) with the amplicon size used to detect TNFα and MxA (<80 bp; Table S1) and found that quantitation of RNA in unfixed and fixed cells was more similar when PCR was performed with a smaller amplicon. This supports the conclusion that fixation reduces the size of the RNA isolated and that quantitation using a larger amplicon size by qPCR can affect accuracy in fixed samples.

### RNA Quantitation using NanoString nCounter® Gene Expression System

**Figure 3:**
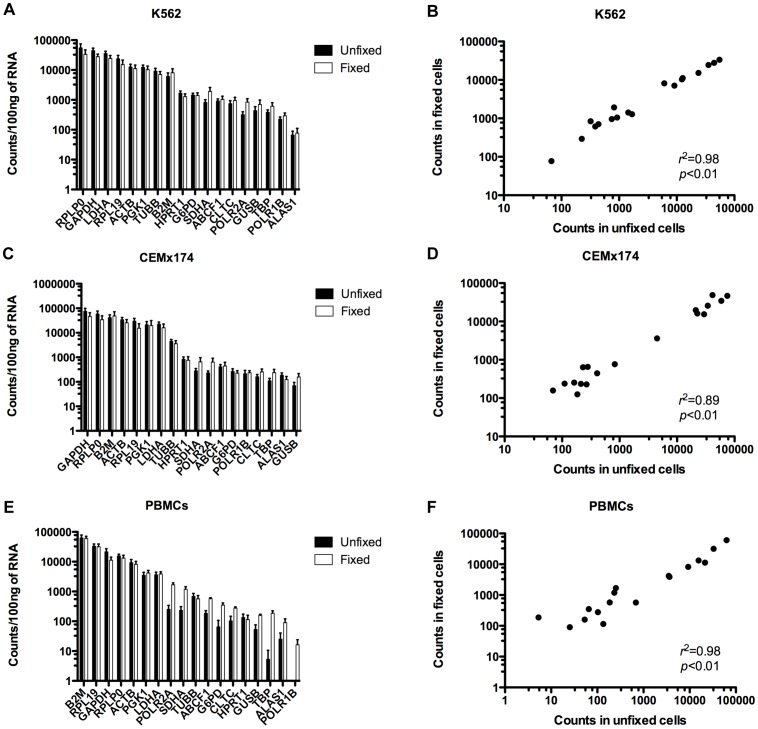
Comparison of mRNA quantitation by NanoString between unfixed and fixed cells. Eighteen human reference gene mRNAs were quantified using NanoString technology in unfixed and fixed samples of K562 (A) and CEMx174 (C) cell lines and human PBMCs (E). Correlation analyses of gene expression levels between unfixed and fixed samples of all cell types are shown (B, D, F). Each point represents one gene and points with zero value were not depicted on graph. Pearson correlation analysis was performed for all data sets. All values shown represent the average value of three biological replicates for unfixed and fixed.

Since we found that fixation of cells with formaldehyde can affect the accuracy of RNA quantitation by RT-qPCR in some cases, we evaluated the efficiency of the NanoString nCounter**®** gene expression system, a probe-based technology that directly measures transcript abundance and does not require cDNA production or amplification [21]–[23]. We used the nCounter**®** Human Reference GX kit to measure 18 human reference genes in the same samples used for RT-qPCR analysis (Figures 3A, C and E). Pearson correlation analyses demonstrated significant correlations of transcript levels between unfixed and fixed samples of K562 and CEMx174 cell lines, and human PBMCs (Figures 3B, D and F) despite RNA degradation in fixed samples. Since the NanoString nCounter**®** system does not require reverse transcription of long intact RNA, this quantitation approach is more reliable for quantitation of gene expression in fixed cells.

### Effect of Sorting and Time after Fixation on RNA Quantitation

**Figure 4:**
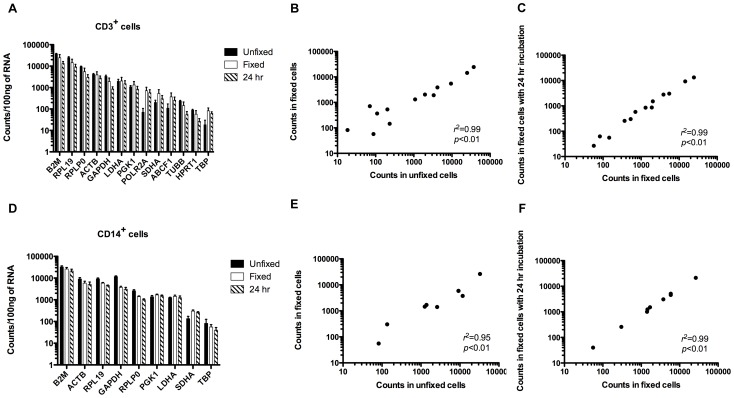
Analysis of the effect of cell sorting and time on mRNA quantitation in fixed cells. Eighteen human reference gene mRNAs were quantified using NanoString technology in unfixed and fixed human CD3^+^ (A) and CD14^+^ (D) cells isolated by FACS. A separate group of fixed cells was incubated at 4°C for 24 hours prior to sorting and RNA isolation. Correlation analyses of gene expression levels between groups are shown for CD3^+^ (B and C) and CD14^+^ (E and F) cells. Each point represents one gene. Pearson correlation analysis was performed for all data sets. All values shown represent the average value obtained from two human donors each containing two technical replicates for unfixed and fixed. Not all genes were included in analysis due to low or undetectable transcript levels.

To evaluate whether the sorting process (potential stress on cells) would alter RNA quantitation in fixed samples, we next collected unfixed and fixed CD3^+^ lymphocytes and CD14^+^ monocytes from human PBMCs sorted by FACS and compared RNA levels using NanoString. Additionally, for a separate group of fixed PBMCs, we performed an overnight incubation at 4°C prior to sorting and RNA isolation to further assess whether time prior to isolation of RNA affected integrity and ability to accurately quantitate the RNA. Figures 4A and D compare transcript quantitation between groups for CD3^+^ and CD14^+^ cells, respectively. Some genes showed low or undetectable transcript levels and were not included in the analysis.

Levels of mRNA in unfixed and fixed CD3^+^ cells showed an excellent correlation (Figure 4B; *r* ^2^ = 0.99). A similar correlation was found when comparing RNA quantitation between fixed cells and cells fixed with a 24-hour incubation prior to sorting (Figure 4C). This suggests that sorting and RNA analysis does not have to be performed immediately following the fixation process.

In the case of CD14^+^ monocytes sorted from PBMCs, comparison of quantitation of genes in unfixed and fixed samples demonstrated a correlation of 0.95 (Figure 4E) while comparison between fixed cells and cells fixed with a 24-hour incubation prior to sorting showed a correlation of 0.99 (Figure 4F). While both correlations were significant (*p*<0.01), the correlation obtained when comparing mRNA quantitation in unfixed cells to that in fixed for CD14^+^ cells (Figure 4E) was lower than that obtained for CD3^+^ cells (Figure 4B). This suggests there may be slight differences in sensitivity to the fixation and sorting process between cell types.

Overall, these results demonstrate that fixation of cells does not alter the quantitation of RNA by NanoString when compared to unfixed primary cells sorted by FACS. Additionally, an incubation time of 24 hours following fixation does not significantly alter RNA quantitation.

### DNA Isolation and Quantitation using qPCR

**Figure 5:**
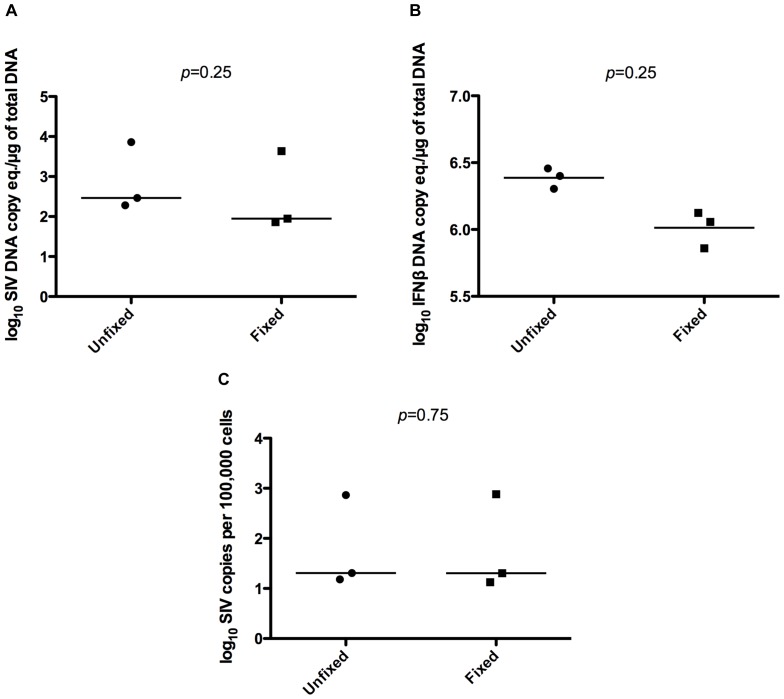
Comparison of SIV DNA quantitation by qPCR between unfixed and fixed cells. SIV (A) and IFNβ (B) DNA copies per total DNA input were measured by qPCR in unfixed and fixed SIV-infected, pigtailed macaque PBMCs from three animals. Calculation of SIV copies per 100,000 cells for each sample was performed using respective IFNβ levels to normalize (C). Each point represents the average value of three technical replicates for one animal. Data were analyzed by Wilcoxon matched-pairs rank test.

We next examined quantitation of DNA in fixed samples by qPCR. Our laboratory studies SIV infection of macaques. During infection, levels of viral DNA can vary among cells types and individuals, and can relate to viral replication and establishment of latent reservoirs [24]; thus, measurement of SIV DNA copies along with a normalizing single-copy cellular gene (IFNβ) is routinely performed. DNA isolation protocols for unfixed and fixed cells were different (see Methods section) as was the case for RNA isolation from unfixed and fixed cells. DNA from unfixed and fixed PBMCs isolated from SIV-infected pigtailed macaques showed no difference in yield between groups (Figure S1). Quantitation of SIV and IFNβ copies per microgram of DNA showed a trend of higher levels in unfixed cells compared to fixed cells although the difference was not significant (Figure 5). Data for individual animals can be found in Figure S2. When the level of the cellular gene (IFNβ) was used to normalize the amount of SIV DNA to the number of SIV copies per 100,000 cells, there was no significant difference in the quantitation of SIV DNA between unfixed and fixed (Figure 5C) and statistical analysis produced a *p* value closer to 1. This demonstrates a proportional decrease in detectable levels of SIV DNA and IFNβ in fixed cells, and normalizes the values for the difference between the fixed and unfixed SIV DNA levels.

## Discussion

We examined the effect of formaldehyde fixation on isolation and quantitation of RNA from cell lines and human primary cells, as well as SIV DNA from macaque PBMCs. We have developed techniques that efficiently isolate nucleic acids from fixed cells and produce yields comparable to those from fresh cells. RNA quantitation in fixed cells by RT-qPCR was reduced by RNA degradation and accurate quantitation was dependent upon PCR amplicon length. Comparison of RNA quantitation between unfixed and fixed samples by the NanoString probe-based system showed strong correlations of transcript levels between fixed and unfixed cells suggesting this technique was more efficient in the quantitation of RNA from fixed cells prepared for FACS analysis. Finally, our results demonstrate that quantitation of SIV DNA in fixed cells by PCR is equivalent to that in unfixed cells when normalization to a cellular gene is performed. Our study has provided validated methods to isolate and accurately quantitate both RNA and DNA in fixed cells analyzed by FACS. These results provide an approach to characterize cell subpopulations by FACS and cell sorting and determine molecular changes in gene expression and viral gene incorporation into cellular DNA.

With our growing knowledge of the existence and importance of cellular subpopulations and the expansion of available antibodies, FACS will continue to serve as an invaluable technique in many fields of research. Fixation of cells prior to FACS analyses preserves cell surface molecules as well as cell integrity and eliminates barriers to the use of fixed cells for further molecular characterization. Although various fixatives exist and others may not be as harsh on nucleic acid integrity, we have chosen formaldehyde because of its ability to preserve cell morphology and cell surface markers, which are features essential to accurate FACS analysis, and due to its common use among laboratories in fixing cells for FACS. Similar to results reported by Reis et al. [15], the NanoString system provided a more reproducible method to quantitate RNA transcripts in fixed cells than RT-qPCR. This is probably due to the reduction in RNA integrity, particularly the size of the RNA fragments, isolated from the cells that were fixed. RNA quantitation using RT-qPCR can be used for fixed cells; however, for optimal results, a minimal target size of the sequence amplified is ideal as shown by our study and previously reported studies [11], [13], [14]. The availability of two techniques, RT-qPCR and NanoString, for transcriptome analysis in fixed cells will prove to be useful in characterizing and determining the function of cellular subpopulations.

For some studies, particularly those involving retroviruses, the quantitation of viral DNA is necessary, and can shed light on cell susceptibility to infection, viral replication and integration, as well as overall disease pathogenesis. SIV and HIV-infected cells are normally handled in Biosafety Level 2 or 3 laboratories and not all laboratories are equipped with sorting equipment; therefore, cells are often fixed so that they can be safely handled and sorted at other facilities. We found that SIV DNA quantitation accuracy is decreased in fixed, macaque PBMCs when compared to unfixed cells and measured by qPCR; however, normalization to a single-copy cellular gene generates SIV copy levels that are equivalent to those quantitated in unfixed cells. This provides an approach to measuring virus DNA in infected cells that are fixed and sorted. Defining which cellular subpopulations in blood and tissue are susceptible to infection and promote the spread of virus in the body is an important step in characterizing viral pathogenesis.

## Supporting Information

Figure S1 **Comparison of RNA and DNA yield between unfixed and fixed cells.** RNA was isolated from unfixed and fixed samples of two cell lines, CEMx174 and K562, and human PBMCs using separate methods. There was no significant difference in RNA yield between unfixed and fixed samples of all cell types (A). DNA was isolated from unfixed and fixed SIV-infected, pigtailed macaque PBMCs by separate methods and no difference in yield was observed between samples (B). Data were analyzed by a two-tailed, paired t test. Statistically significant *p* values are shown.
(TIF)

Figure S2 Comparison of SIV DNA quantitation by qPCR between unfixed and fixed PBMCs from three SIV-infected pigtailed macaques. SIV (A) and IFNβ (B) DNA copies per total DNA input were measured by qPCR in unfixed and fixed SIV-infected, pigtailed macaque PBMCs from three animals. Calculation of SIV copies per 100,000 cells for each sample was performed using respective IFNβ levels to normalize (C). Data for individual animals are shown and three technical replicates are reported for each animal.
(TIF)

Table S1 **Amplicon lengths for RT-qPCR reactions.**
(PDF)

Table S2 **Primer and probe sequences for qPCR reactions.**
(PDF)

Table S3 **nCounter® Human Reference GX kit probe set.**
(PDF)
