# Supplementary figures and images for: Quantitation of Gene Expression in Formaldehyde-Fixed and Fluorescence-Activated Sorted Cells
Source: PLoS One. 2013 Sep 2;8(9):e73849. doi: 10.1371/journal.pone.0073849 (PMC3759445; doi:10.1371/journal.pone.0073849)

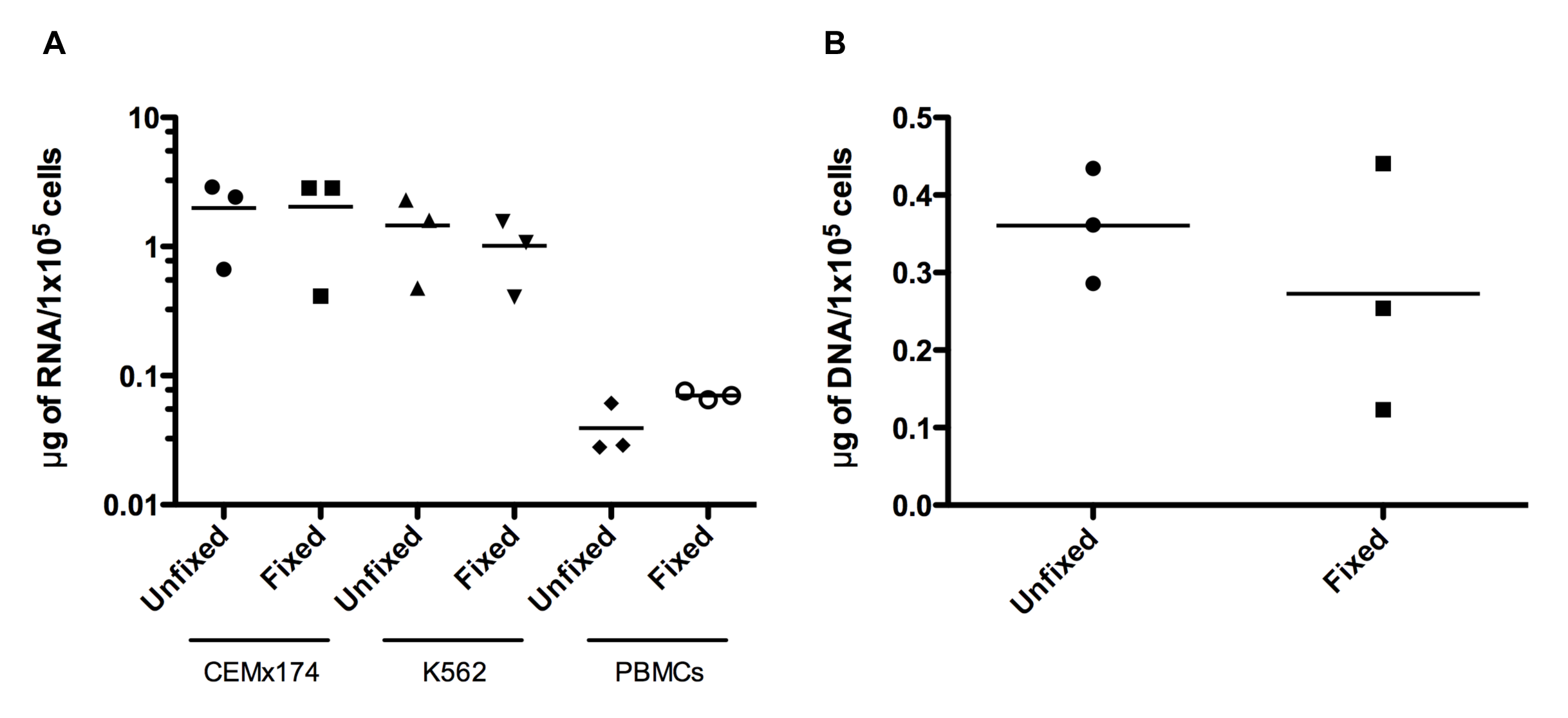

Supplement: Figure S1 — Comparison of RNA and DNA yield between unfixed and fixed cells. RNA was isolated from unfixed and fixed samples of two cell lines, CEMx174 and K562, and human PBMCs using separate methods. There was no significant difference in RNA yield between unfixed and fixed samples of all cell types (A). DNA was isolated from unfixed and fixed SIV-infected, pigtailed macaque PBMCs by separate methods and no difference in yield was observed between samples (B). Data were analyzed by a two-tailed, paired t test. Statistically significant p values are shown. (TIF) [file pone.0073849.s001.tif]

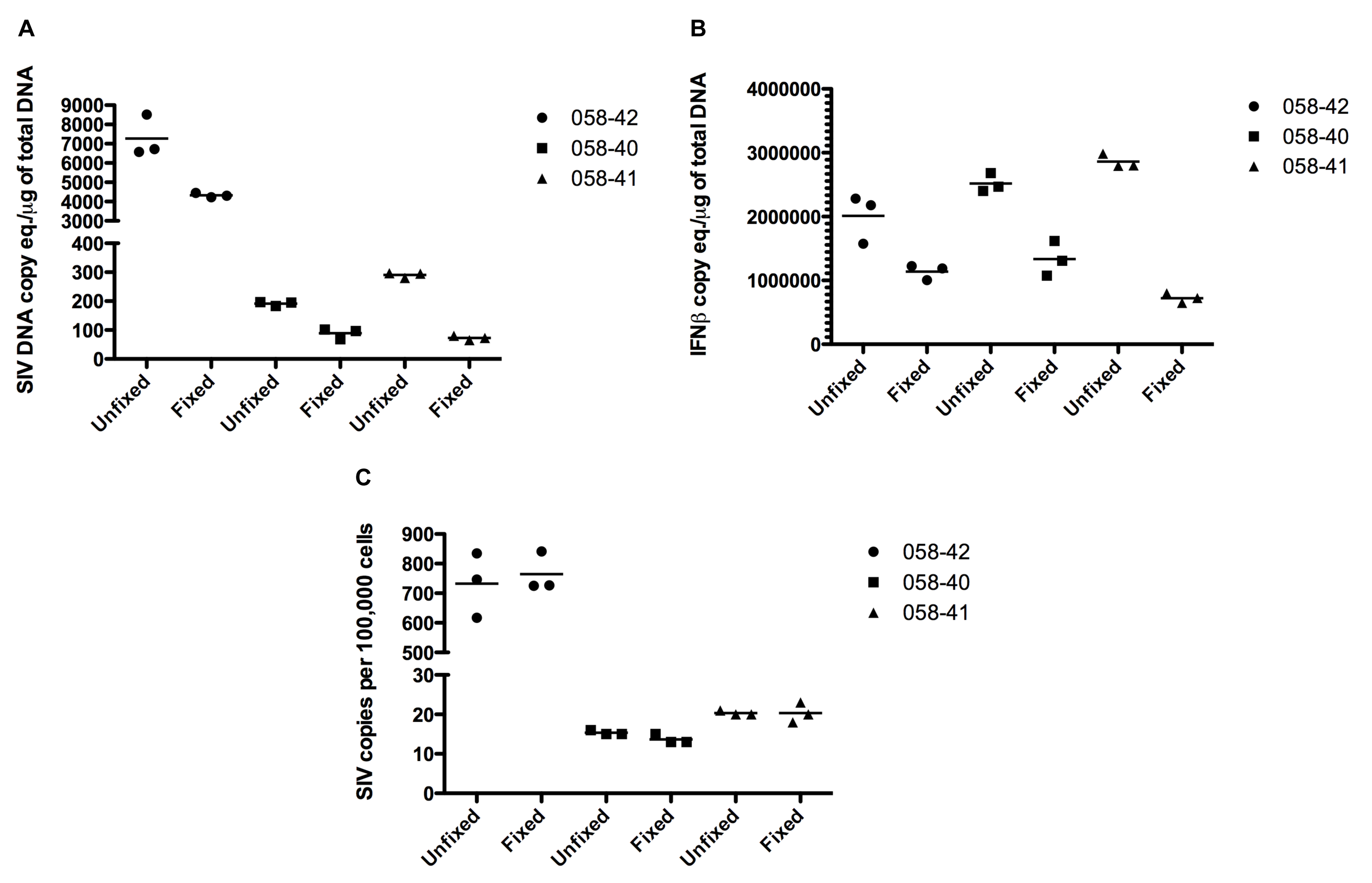

Supplement: Figure S2 — Comparison of SIV DNA quantitation by qPCR between unfixed and fixed PBMCs from three SIV-infected pigtailed macaques. SIV (A) and IFNβ (B) DNA copies per total DNA input were measured by qPCR in unfixed and fixed SIV-infected, pigtailed macaque PBMCs from three animals. Calculation of SIV copies per 100,000 cells for each sample was performed using respective IFNβ levels to normalize (C). Data for individual animals are shown and three technical replicates are reported for each animal. (TIF) [file pone.0073849.s002.tif]
