# Supplementary material for: Quantitation of Gene Expression in Formaldehyde-Fixed and Fluorescence-Activated Sorted Cells
Source: PLoS One. 2013 Sep 2;8(9):e73849. doi: 10.1371/journal.pone.0073849 (PMC3759445; doi:10.1371/journal.pone.0073849)
Supplement: Table S1 — Amplicon lengths for RT-qPCR reactions. (PDF) [file pone.0073849.s003.pdf]

Table S1. Amplicon lengths for RT-qPCR reactions.

Gene	Amplicon size (bp)
18S	104
GAPDH	111
TNFα	75
MxA	78
