# Supplementary material for: Quantitation of Gene Expression in Formaldehyde-Fixed and Fluorescence-Activated Sorted Cells
Source: PLoS One. 2013 Sep 2;8(9):e73849. doi: 10.1371/journal.pone.0073849 (PMC3759445; doi:10.1371/journal.pone.0073849)
Supplement: Table S2 — Primer and probe sequences for qPCR reactions. (PDF) [file pone.0073849.s004.pdf]

**Table S2. Primer and probe sequences for qPCR reactions.**

| Gene | Forward Primer | Reverse Primer | Probe |
|---|---|---|---|
| 18S | TAG AGG GAC AAG TGG CGT TC | CGC TGA GCC AGT CAG TGT | AGC AAT AAC AGG TCT GTG ATG |
| GAPDH | AGC CTC AAG ATC ATC AGC AAT G | ATG GAC TGT GGT CAT GAG TCC TT | CCA ACT GCT TAG CAC CCC TGG CC |
| TNFα | GGC TCA GGC AGT CAG ATC ATC | GCT TGA GGG TTT GCT ACA ACA TG | TCG AAC CCC AAG TGA CAA GCC TGT AGC |
| MxA | AGG AGT TGC CCT TCC CAG A | TCG TTC ACA AGT TTC TTC AGT TTC A | ACC AGC GGG CAT CTG GTC ACG A |
| SIV | GTC TGC GTC ATC TGG TGC ATT C | CAC TAG GTG TCT CTG CAC TAT CTG TTT TG | CTT CCT CAG TGT GTT TCA CTT TCT CTT CTG |
| IFNβ | GCC TCA AGG ACA GGA TGA ACT T | GCG TCC TCC TTC TGG AAC TG | CAT CCC TGA GGA AAT TAA GCA GCC GC |
