# Supplementary material for: Quantitation of Gene Expression in Formaldehyde-Fixed and Fluorescence-Activated Sorted Cells
Source: PLoS One. 2013 Sep 2;8(9):e73849. doi: 10.1371/journal.pone.0073849 (PMC3759445; doi:10.1371/journal.pone.0073849)
Supplement: Table S3 — nCounter® Human Reference GX kit probe set. (PDF) [file pone.0073849.s005.pdf]

Table S3. nCounter® Human Reference GX kit probe set.

Gene	Accession Number	Sequence
ABCF1	NM_001090.2	GATGTCCTCCCGCCAAGCCATGTTAGAAAATGCATCTGACATCAAGCTGGAGAAGTTCAGCATCTCCGCTCATGGCAAGGAGCTGTTCGTCAATGCAGAC
ACTB	NM_001101.2	TGCAGAAGGAGATCACTGCCCTGGCACCCAGCACAATGAAGATCAAGATCATTGCTCCTCCTGAGCGCAAGTACTCCGTGTGGATCGGCGGCTCCATCCT
ALAS1	NM_000688.4	GGGGATCGGGATGGAGTCATGCCAAAAATGGACATCATTTCTGGAACACTTGGCAAAGCCTTTGGTTGTGTTGGAGGGTACATCGCCAGCACGAGTTCTC
B2M	NM_004048.2	CGGGCATTCCTGAAGCTGACAGCATTCGGGCCGAGATGTCTCGCTCCGTGGCCTTAGCTGTGCTCGCGCTACTCTCTCTTTCTGGCCTGGAGGCTATCCA
CLTC	NM_004859.2	GGGTATCAACCCAGCAAACATTGGCTTCAGTACCCTGACTATGGAGTCTGACAAATTCATCTGCATTAGAGAAAAAGTAGGAGAGCAGGCCCAGGTGGTA
G6PD	NM_000402.2	ACAACATCGCCTGCGTTATCCTCACCTTCAAGGAGCCCTTTGGCACTGAGGGTCGCGGGGGCTATTTCGATGAATTTGGGATCATCCGGGACGTGATGCA
GAPDH	NM_002046.3	TCCTCCTGTTCGACAGTCAGCCGCATCTTCTTTTGCGTCGCCAGCCGAGCCACATCGCTCAGACACCATGGGGAAGGTGAAGGTCGGAGTCAACGGATTT
GUSB	NM_000181.1	CGGTCGTGATGTGGTCTGTGGCCAACGAGCCTGCGTCCCACCTAGAATCTGCTGGCTACTACTTGAAGATGGTGATCGCTCACACCAAATCCTTGGACCC
HPRT1	NM_000194.1	TGTGATGAAGGAGATGGGAGGCCATCACATTGTAGCCCTCTGTGTGCTCAAGGGGGGCTATAAATTCTTTGCTGACCTGCTGGATTACATCAAAGCACTG
LDHA	NM_005566.1	CAGAATGGAATCTCAGACCTTGTGAAGGTGACTCTGACTTCTGAGGAAGAGGCCCGTTTGAAGAAGAGTGCAGATACACTTTGGGGGATCCAAAAGGAGC
PGK1	NM_000291.2	GCAAGAAGTATGCTGAGGCTGTCACTCGGGCTAAGCAGATTGTGTGGAATGGTCCTGTGGGGGTATTTGAATGGGAAGCTTTTGCCCGGGGAACCAAAGC
POLR1B	NM_019014.3	GGAGAACTCGGCCTTAGAATACTTTGGTGAGATGTTAAAGGCTGCTGGCTACAATTTCTATGGCACCGAGAGGTTATATAGTGGCATCAGTGGGCTAGAA
POLR2A	NM_000937.2	TTCCAAGAAGCCAAAGACTCCTTCGCTTACTGTCTTCCTGTTGGGCCAGTCCGCTCGAGATGCTGAGAGAGCCAAGGATATTCTGTGCCGTCTGGAGCAT
RPL19	NM_000981.3	CCAATGCCCGAATGCCAGAGAAGGTCACATGGATGAGGAGAATGAGGATTTTGCGCCGGCTGCTCAGAAGATACCGTGAATCTAAGAAGATCGATCGCCA
RPLP0	NM_001002.3	CGAAATGTTTCATTGTGGGAGCAGACAATGTGGGCTCCAAGCAGATGCAGCAGATCCGCATGTCCCTTCGCGGGAAGGCTGTGGTGCTGATGGGCAAGAA
SDHA	NM_004168.1	TGGAGGGGCAGGCTTGCGAGCTGCATTTGGCCTTTCTGAGGCAGGGTTTAATACAGCATGTGTTACCAAGCTGTTTCCTACCAGGTCACACACTGTTGCA
TBP	NM_003194.3	CGCCGGCTGTTTAACTTCGCTTCCGCTGGCCCATAGTGATCTTTGCAGTGACCCAGCAGCATCACTGTTTCTTGGCGTGTGAAGATAACCCAAGGAATTG
TUBB	NM_178014.2	TGGTGGATCTAGAACCTGGGACCATGGACTCTGTTCGCTCAGGTCCTTTTGGCCAGATCTTTAGACCAGACAACTTTGTATTTGGTCAGTCTGGGGCAGG
